# Overview and characterization of penile cancer content across social media platforms

**DOI:** 10.3389/fonc.2023.1301973

**Published:** 2023-12-11

**Authors:** Ruben Alejandro Ortiz-Guerra, Salvador Jaime-Casas, Bertha Alejandra Martinez-Cannon, Jose C. Ariza-Avila, Ana P. González-Morales, Andrea Bardan-Duarte, Yuly A. Remolina-Bonilla, Philippe E. Spiess, Maria T. Bourlon

**Affiliations:** ^1^ Department of Hematology and Oncology, Instituto Nacional de Ciencias Medicas y Nutricion Salvador Zubiran, Mexico City, Mexico; ^2^ School of Medicine, Universidad Panamericana, Mexico City, Mexico; ^3^ Department of Genitourinary Oncology, Moffitt Cancer Center, Tampa, FL, United States

**Keywords:** social media, penile cancer, platforms, posts, content

## Abstract

**Background:**

Social media platforms (SMP) are an emerging resource that allows physicians, patients, and families to converse on cancer prevention, diagnosis, and treatment. We aimed to characterize penile cancer (PC) content shared on SMP.

**Methods:**

We searched PC posts on Twitter, Facebook, and Instagram from July 1st, 2021, through June 30th, 2022. Two independent, blinded reviewers analyzed the hashtags: #PenileCancer, #PenileCancerAwareness, and #PenileNeoplasm. Descriptive statistics were used for posts characterization, Pearson´s correlation coefficient for associations, and Cohen’s weighted kappa coefficient for inter-rater agreement rate.

**Results:**

A total of 791 posts were analyzed, with Twitter accounting for 52%, Facebook for 12.2%, and Instagram for 35.5%, and. Most posts originated from high-income countries, such as the United Kingdom (48.8%). We found no correlation between the number of posts with PC incidence (p = 0.64) or users on SMP (p = 0.27). Most accounts were classified as “support and awareness communities” (43.6%) and “physicians and clinical researchers” (38.2%). Urology was the most common medical specialty to post (60.9%), followed by oncology (11.3%). Most posts were classified as “prevention and awareness for users” (45.1%). Global inter-reviewer agreement rate was almost perfect (k=0.95; p ≤ 0.01). On Twitter, “physicians and clinical researchers” shared more content on “treatment updates and medical papers published in medical journals,” while on Facebook and Instagram, “support and awareness communities” focused on “personal and support comments.”

**Conclusion:**

Overall, the number of PC posts was low compared to other neoplasms across the SMP evaluated in this study. “Physicians and clinical researchers” shared more content on Twitter, while “support and awareness communities” on Facebook and Instagram. Encouraging the use of a common SMP among the medical community and general users could lead to a more effective communication between physicians, patients, and support groups, and to increased awareness of PC.

## Introduction

Penile cancer (PC) is a rare neoplasm with a prevalence of 0.1 - 1 per 100,000 men in high-income countries (1). However, its prevalence in low- and middle-income countries in some African, Asian, and South American regions reaches as high as 20% of all male malignancies (1, 2). PC affects predominantly men in the 6^th^ and 7^th^ decade of life (3), and human papillomavirus (HPV) infection is recognized as the leading risk factor for this disease (4). Additional risk factors include smoking, local trauma, and poor hygiene; most of which are preventable in the first decades of life, when exposure is the highest (4). Conversely, circumcision is described as a protective factor against PC (3). At diagnosis, 30-60% of patients will present with locally advanced disease (5) and approximately 10% with distant metastasis (6). Since most patients will have a more advanced stage at diagnosis, current treatment modalities result in devastating disfigurement of the genital area, sexual dysfunction, and psychosexual distress, all contributing to a significant decrease in quality of life (7).

Social media has been shown to improve patient’s access to healthcare information (8). Furthermore, physicians are also using social media to promote patient healthcare education regarding multiple diseases, including cancer (9). Social media platforms (SMP) are online forums that allow users to create, share, comment, and modify content (10). Facebook, Instagram, and Twitter (currently X) are the most popular SMP, with 2.9 billion, 1.6 billion, and 372 million active users worldwide by April 2023, respectively (11). Multiple oncology-specific online communities have rapidly spurred, with breast cancer social media (#BCSM), lung cancer social media (#LCSM), gynecologic cancer social media (#GYNCSM), and multiple myeloma social media (#MMSM) being notable examples (12). These hashtags demonstrate a precedent for online discussions between providers and patients, and this phenomenon could be extended to other neoplasms (13, 14).

Although previous Twitter analysis studies focusing on information exchange patterns have been published regarding other neoplasms such as breast and prostate cancer (15), to date, no prior reports have analyzed PC content posted across different SMP. Therefore, this study aimed to characterize PC-related content shared on SMP, determine the demographics of the posts, and evaluate interactions and engagement.

## Materials and methods

In this observational study, we searched for posts regarding PC on three SMP (Twitter [currently X], Facebook, and Instagram) from July 1st, 2021, through June 30th, 2022. Original posts with the specific hashtags of interest (#PenileCancer, #PenileCancerAwareness, and #PenileNeoplasm) in any language, posted from any geographical location, from any type of account (verified account, personal account, group account), and with any kind of content format (URL links, text, image, video, GIF’s) were included.

Search engines were configured to include sensitive posts to broaden our data pool. We did not use any pre-coded search algorithm software. Our search strategy for all three platforms used an intelligent algorithm through the advanced search option. Posts from accounts with low activity (<50 posts or <100 followers) were excluded to avoid bots and fake accounts (16). Posts from both verified and non-verified accounts were included in our analysis. Posts were excluded if duplicated or sent as replies to other accounts. Each post was individually analyzed, and two independent blinded reviewers manually coded relevant information.

The users’ country of origin was documented and classified based on the 2021 World Bank Data group classification (17). Posts’ content was classified into 1) “prevention and awareness for users,” 2) “treatment updates and medical papers published in medical journals,” 3) “medical meetings and networking among healthcare providers,” 4) “support and personal comments” and 5) “miscellaneous or general users.” We recorded the number of followers and following accounts each user had. Accounts were categorized as 1) “physicians and clinical researchers,”; 2) “other healthcare professionals”; 3) “healthcare centers,”; 4) “support and awareness communities”; 5) “medical journals,”; 6) “news media companies”; 7) “general users and patients”. The medical specialty of “physicians and clinical researchers” was also recorded if available. Categories for users and types of content were based on previously published studies (18–22).

Inter-rater agreement was calculated with Cohen’s weighted kappa coefficient. Public metrics for each post (number of shares, likes, comments) were collected to assess their degree of interaction. Descriptive statistics were used for post characterization (number of posts, origin, content, type of account). Pearson´s correlation coefficient was used to evaluate the association between the number of posts, region, and incidence of PC. A p-value <0.05 was considered statistically significant. The software used for data analysis was SPSS version 25 (SPSS, Chicago, Illinois).

## Results

### Posts’ demographics

A total of 1168 posts regarding PC were identified on Facebook, Instagram, and Twitter. After eliminating duplicated posts, a total of 791 were coded and analyzed. Among these, 411 (52%) were obtained from Twitter, 99 (12.5%) from Facebook, and 281 (35.5%) from Instagram. Posts were written in eight languages, most in English (82.7%, n = 654), followed by Spanish (14.7%, n = 116).

A total of 311 accounts were identified. Gender could be determined for 24.6% of Twitter accounts, with 19.7% from male users and 4.9% from female users. From Facebook, gender could be determined for 20.2% of accounts, with 19.2% from male users and 5.1% from female users.

As for Instagram, gender could be determined in 51.6% of accounts, with 38.4% from male users and 13.2% from female users.

**Figure 1:**
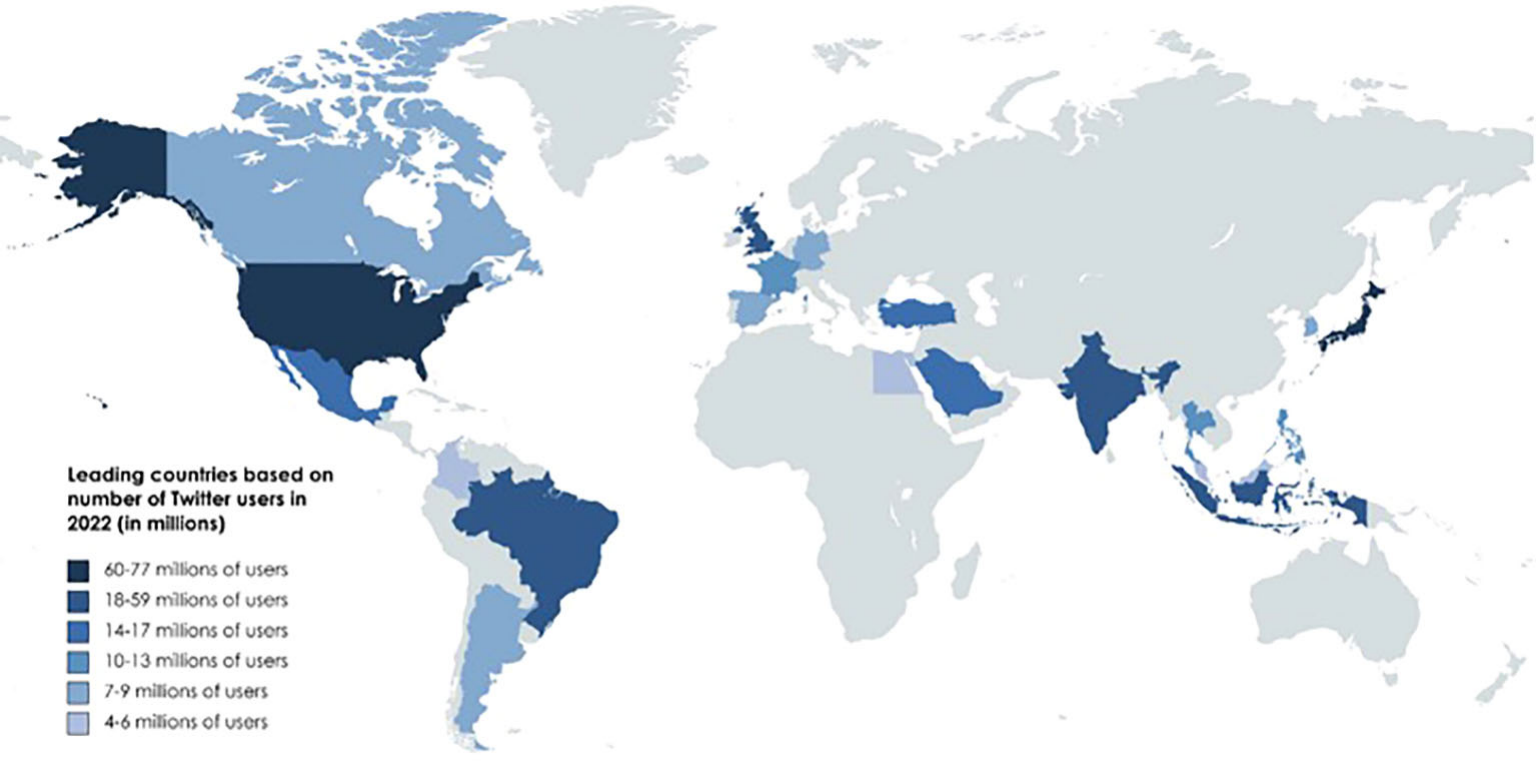
World map distribution of penile cancer posts.

The accounts’ country of origin was identified in 93.3% of cases, with 28 countries documented. The most prevalent countries of origin were the United Kingdom (UK) (48.8%, n = 386), the United States of America (USA) (15.3%, n = 121), Mexico (5.4%, n = 43) and India (5.1%, n = 40). According to the World Bank, posts originated in high, upper-middle, and lower-middle-income countries in 82.3%, 10.5%, and 7.1% of cases, respectively (17). Worldwide post distribution is shown in **Figure 1**. Pearson’s correlation coefficient showed that the number of posts was not correlated to PC incidence in the region of origin (p = 0.64) or the total number of users on SMP (p = 0.27).

**Figure 2:**
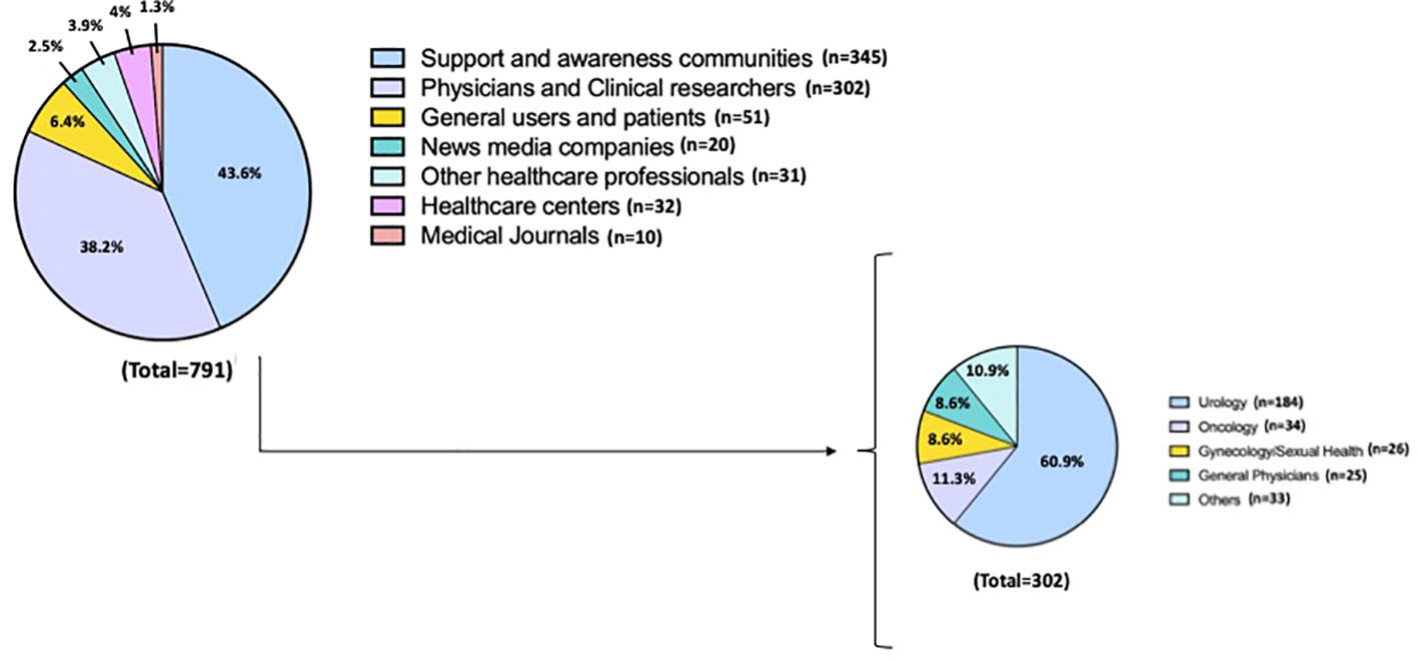
Overall accounts categories.

Most accounts belonged to the “support and awareness” (43.6%, n = 345) and “physicians and clinical researchers” (38.2%, n = 302) category. Among the “physicians and clinical researchers” subgroup (n=302), the most identified medical specialty was “urology” (60.9%, n = 184), followed by “oncology” (11.3%, n = 34) and “gynecologists/sexual health specialists” (8.6%, n = 26). Account categories according to SMP are shown in **Figure 2**. Verified accounts (6.7%) posted only 12.5% of the total content. Most verified accounts belonged to “medical journals” (45.3%) and “influencers” (23.4%).

### Interactions and engagement

**Figure 3:**
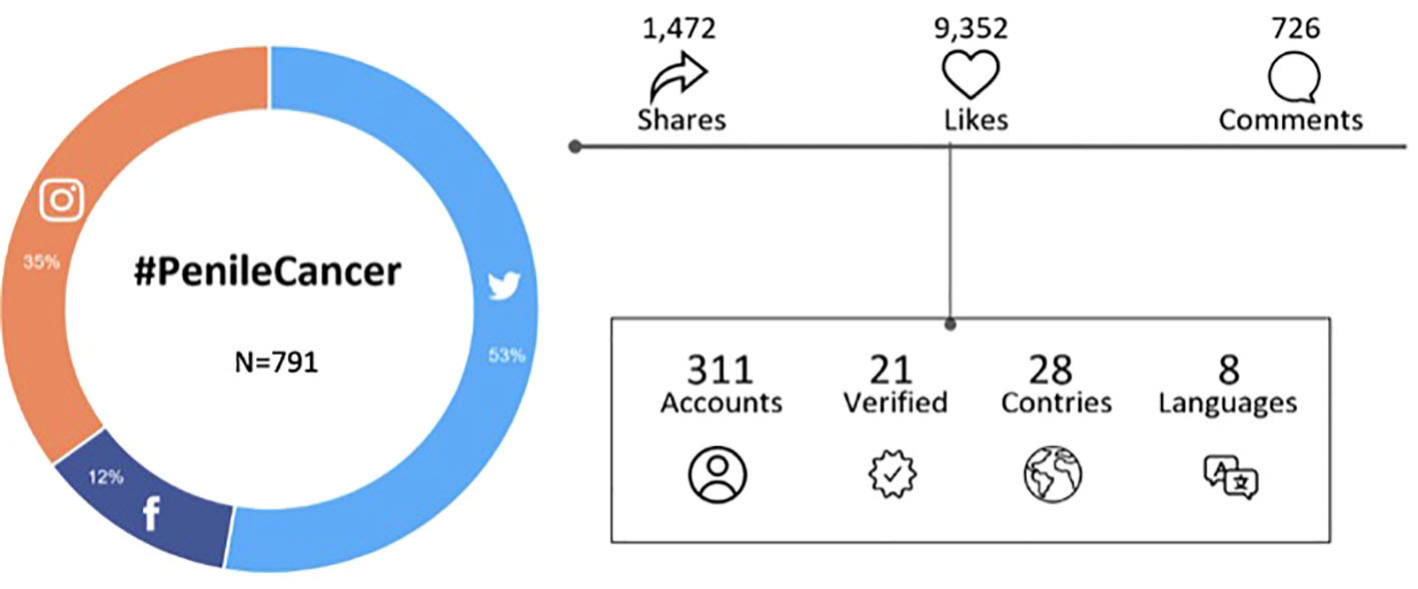
Interactions and engagement.

Among the 791 posts included in this analysis, 9,352 likes, 726 comments, and 1472 shares/retweets were identified (**Figure 3**). The median number of likes was 7 (range 0-299), the median number of comments was 1 (range 0-84), and the median number of shares/retweets was 3 (range 0-111). A total of 414 (51.8%) posts have at least one interaction (like, share, or comment), 46 (5.6%) have more than five comments, 154 (18.5%) have more than five shares/retweets, and 182 (50.1%) have more than five likes. When adjusted by platform the median number of posts per month was 34 (range 18-57), 8 (range 1-48), and 20 (range 13-46), on Twitter, Facebook, and Instagram, respectively.

### Content analysis

Most posts were about “prevention and awareness for users” (N = 357; 45.1%), “support and personal comments” (N = 200; 25.3%), “treatment updates and medical papers published in medical journals” (N = 133; 16.8%), and “medical meetings and networking among healthcare providers” (N = 90; 11.4%). Content across SMP can be seen in **Table 1**. The type of content was classified for all posts by two independent blinded reviewers, with an inter-reviewer global agreement was almost perfect (k=0.95; p ≤ 0.01). The inter-rater agreement was k=0.95 (p ≤ 0.01), k=0.87 (p ≤ 0.05), and k=0.91 (p ≤ 0.05), for Twitter, Facebook, and Instagram, respectively. Agreement was higher for posts concerning “prevention and awareness for users” (k=0.93), “treatment updates and medical papers published in medical journals” (k=0.89), and lower for “medical meetings and networking among healthcare providers” (k=0.86).

**Table 1:** Overall content across SMP.

Content type	Overall (n = 791)		Twitter (n = 428)		Facebook (n = 99)		Instagram (n = 281)	
	n	%	n	%	n	%	n	%
Prevention and awareness	357	45.1	151	57.3	45	45.5	161	57.3
Support and personal comments	200	25.3	78	19.0	30	30.3	92	32.7
Treatment updates and medical papers published in medical journals	133	16.8	108	26.3	7.0	7.1	18	6.4
Medical meetings and networking among healthcare providers	90	11.4	74	11	11.1	11.6	5.0	1.8
Not classifiable	11	1.4	0	0	6.0	6.1	5.0	1.8

On Twitter, most content was also shared by “support and awareness communities” (n = 218, 53%), followed by “physicians and clinical researchers” (34.1%, n = 140). On Facebook, most content was posted by “support and awareness communities” (n = 49, 49.5%), while on Instagram, content was most frequently shared by “physicians and clinical researchers” (n = 129, 45.9%). Among the “support and awareness communities” category, most of the content on Twitter was about “prevention and awareness for users” (48.6%, n = 106), as for Facebook and Instagram, posts were about “support and personal comments” in 51% (n = 25) and 59% (n = 46), respectively. Content shared by physicians on Twitter was most often about “treatment updates and medical papers published in medical journals” (57.9%, n = 81) and “medical meetings and networking among healthcare providers” (24.3%, n = 34). On Facebook and Instagram, the same account category posted content regarding “prevention and awareness for users” in 60.6% (n = 20) and 82.2% (n = 106) of cases, respectively.

## Discussion

Our study provides an overview of penile cancer-related content across different SMP over a period of 12 months. In 2016, Borgmann et al. described content related to other genitourinary neoplasms across different SMP, but to our knowledge, PC was not included (13, 23). Currently, the Cancer Tag Ontology (CTO) project has not included a disease-specific, short, and unique hashtag for PC, nor have international guidelines or medical societies endorsed recommendations on search algorithms for PC-related content on SMP (24). Although penile cancer is a low-prevalence disease, the creation specific hashtags even for rare diseases such #MPNSM has been proven to raise awareness for this condition and to feature a greater variety of voices aside from academic leaders (25).

Furthermore, users’ age could have also contributed to the low number of PC-related posts across the different SMP. Individuals in their 50s and 60s are less likely to participate and share content across SMP actively (26).In contrast, their younger counterparts are more likely to share their experiences and utilize SMP as a means of networking with other individuals that share similar interests (27–29). Since PC presents most commonly in men in the 6^th^ and 7^th^ decade of life, patients with PC represent the age group less likely to utilize SMP for obtaining and sharing information (28). Conversely, as users among SMP tend to be younger, PC-related content could prove most impactful among this audience by disseminating information concerning healthy habits and modifying risk factors to decrease PC incidence in later decades of life (29–31). Although PC is a low-prevalence disease, the creation specific hashtags even for rare diseases such #MPNSM has been proven to raise awareness for this condition and to feature a greater variety of voices aside from academic leaders (25).

Notably, most posts originated from the UK and USA, both of which are countries with a very low prevalence and incidence of PC (1). These and other high-income countries are at the forefront of preventive medicine with a stronger promotion of safe sex practices, which could explain why disease prevalence is also the lowest compared to less developed nations (32). On the contrary, low-income countries, such as those in Latin America and the Middle East, are less active across different SMP and have a higher PC burden (33). However, we did not find a correlation between the number of posts and disease incidence or users per region. Hence, low SMP engagement from low-income countries could be explained by income level and barriers to access to healthcare, internet access, education level, and a lack of governmental or social campaigns for cancer prevention and awareness.

“Physicians and clinical researchers” had significantly more medical-related activity such as “treatment updates and medical papers published in medical journals” on Twitter than on Instagram and Facebook, which resembles the increased popularity of the former platform among healthcare professionals (34). “Physicians and clinical researchers” also shared content focused on “networking among healthcare providers,” which compliments previously reported data on healthcare providers utilizing SMP for professional development and networking (35). As an example, in 2015, the Collaborative for Social Media Outcomes in Oncology (COSMO) was founded by a group of oncology professionals who met on Twitter and gathered to guide appropriately managing social media while always considering patients’ right to privacy and best interest at hand (9).As online tools become increasingly used by healthcare providers on a daily basis, further dissemination and awareness of currently available recommendations is necessary to ensure the responsible use of SMP among oncology specialists.

In contrast, we found that “support and awareness communities” utilized Facebook and Instagram more frequently than Twitter to share PC-related content. This may be partly explained by the more interactive and user-friendly interfaces of Instagram and Facebook (36). We hypothesize that this difference in SMP preference further hinders optimal patient-physician communication and represents a missed opportunity for a common platform where interaction between all parties could be more efficient. This is particularly important, as 62% of social media users reported having a higher level of trust for information provided by physicians on these platforms as compared to the same information provided by non-social media websites, family, and friends (10). Thus, the potential impact of patient and physician engagement within the same SMP is yet to be exploited.

Our study is limited by the need for a standardized method to analyze SMP content formally. Online content differs substantially among different time frames and other cancer campaigns could have potentially overshadowed PC-related content during our search period (37). Furthermore, we only focused on hashtag-specific content, which may have resulted in the loss of posts with a more general content scope (38). Social media search engines may not retrieve all available content due to limitations on specific timeframes, differences in relevance algorithms, and banning sensitive content. Although Facebook, Twitter, and Instagram stand out as top social media in many countries, we acknowledge restricting our study to these platforms may lead to selection bias by excluding certain SMPs that are specific to certain regions. However, these SMP were chosen for our study because they share three common features: 1) enable users to create a personal profile, 2) facilitate the creation of a list of online connections, and 3) offer the capability to view and interact with a continuously updated information feed that includes posts from one’s online connections. Finally, we acknowledge that age, education, income, and gender are also important factors influencing SMP use across countries *(* 26 *)*, thus limiting the generalization of our findings. As considered in our search methodology, we tried to avoid bias by classifying posts by two independent blinded reviewers with an almost perfect inter-rater agreement. Using the CTO directives, we aimed to use the most up-to-date and frequently used hashtags among SMP for PC-content. Additionally, the exclusion of users that share content with keywords in other languages or with spelling mistakes may have led to further selection bias. We also acknowledge that age, education, income, and gender are also important factors influencing SMP use across countries, thus limiting the generalization of our findings (26). We sought to diminish these limitations by expanding the timeframe of posts searched to 12 months. This enabled us to collect data with a more realistic scope of PC-related activity across these platforms. This was a significantly longer timeframe than used in similar studies regarding other neoplasms that considered only days to months for their analyses (39–41). Finally, our study represents the first to evaluate the number of posts and characterization of content related to PC on SMPs, thus, our findings show the current status of PC-related discussions and gives reference for future comparisons.

Further research is warranted to determine social media’s impact on preventive health behaviors, oncological outcomes, and patient’s quality of life. Although cancer campaigns on social media have made great efforts to encourage public engagement, multiple opportunities still exist for improving physician-patient communication, dissemination of evidence-based medical information, and inclusion of other medical specialties in the discussion. SMP represents an opportunity for sharing holistic, fact-checked information for multiple neoplasms, however, caution must be exercised as misinformation could hinder progress in patient care.

## Conclusion

Overall, the number of PC posts was low across the SMP evaluated in this study, with Twitter being the SMP most frequently used for PC-related content. The most prevalent countries of origin were high-income countries such as the UK and USA, however, the prevalence of PC is relatively low in these regions. Most accounts belonged to the “support and awareness communities” category, followed by “physicians and clinical researchers.” Nonetheless, certain differences were observed on how these account categories use SMP. “Physicians and clinical researchers” shared more content on Twitter, while “support and awareness communities” on Facebook and Instagram. Hence, the full potential of patient and physician interaction within the same SMP remains to be explored.

Further research is warranted to determine social media’s impact on patients’ information-seeking behaviors, oncological outcomes, and quality of life. Multiple opportunities still exist for improving physician-patient communication, disseminating evidence-based medical information, and including other medical specialties in the discussion. Encouraging the use of a common SMP among the medical community and general users could lead to a more effective communication between physicians, patients, and support groups, and to increased awareness of PC globally.

## Data availability statement

The raw data supporting the conclusions of this article will be made available by the authors, without undue reservation.

## Author contributions

RAOG: Formal Analysis, Writing – original draft, Writing – review & editing. SJC: Formal Analysis, Writing – original draft, Writing – review & editing. BAMC: Writing – original draft, Writing – review & editing, Formal Analysis. JCAA: Writing – review & editing. APGM: Writing – review & editing. ABD: Writing – original draft, Data curation, Methodology, Supervision, Conceptualization. YARB: Writing – review & editing. PES: Writing – review & editing. MTB: Conceptualization, Methodology, Supervision, Writing – original draft, Writing – review & editing.
